# Nursing Activities Score in critically ill patients on invasive mechanical ventilation

**DOI:** 10.1590/0034-7167-2025-0390

**Published:** 2026-10-19

**Authors:** Larissa Moreira Bianchi, Tayse Tâmara da Paixão Duarte, Michelle Zampieri Ipolito, Kamilla Grasielle Nunes da Silva, Walterlânia Silva Santos, Marcia Cristina da Silva Magro

**Affiliations:** I Universidade de Brasília, Faculdade de Ciências e Tecnologia em Saúde. Brasília, Distrito Federal, Brazil; II Universidade de Brasília, Hospital Universitário de Brasília. Brasília, Distrito Federal, Brazil

**Keywords:** Respiration, Artificial, Workload, Nursing, Organ Dysfunction Scores, Critical Care., Respiración Artificial, Carga de Trabajo, Enfermería, Puntuaciones en la Disfunción de Órganos, Cuidados Críticos.

## Abstract

**Objective:**

to assess the association between invasive mechanical ventilation duration, classified as prolonged and non-prolonged, and nursing workload in critically ill patients.

**Method:**

a prospective cohort study with adult patients on mechanical ventilation for up to 21 days or more. Nursing workload was measured using the Nursing Activities Score (NAS). Clinical data were collected using a structured questionnaire. A significance level of 5% was adopted.

**Results:**

considering 61 patients, there was no significant difference in nursing workload between prolonged and non-prolonged mechanical ventilation according to NAS [111.2 (105.118) vs. 111.8 (107.119); p=0.880], nor was there an association between mechanical ventilation time and this score (AUC=0.512). Prolonged mechanical ventilation was associated with vasoactive drugs (96%; p=0.007) and greater severity of acute kidney injury (KDIGO=3.78%; p=0.009).

**Conclusion:**

mechanical ventilation duration was not associated with changes in nursing workload, which remained high regardless of the ventilatory support duration.

## RESUMEN

**Objetivo:**

evaluar la asociación entre la duración de la ventilación mecánica invasiva, clasificada como prolongada o no prolongada, y la carga de trabajo de enfermería en pacientes críticos.

**Método:**

estudio de cohorte prospectivo con pacientes adultos sometidos a ventilación mecánica durante 21 días o más. La carga de trabajo de enfermería se midió mediante el Nursing Activities Score (NAS). Los datos clínicos se recopilaron mediante un cuestionario estructurado. Se adoptó un nivel de significancia del 5%.

**Resultados:**

considerando 61 pacientes, no hubo diferencia significativa en la carga de trabajo de enfermería entre la ventilación mecánica prolongada y la no prolongada según la escala NAS [111,2(105,118)vs.111,8(107,119);p=0,880], ni hubo asociación entre el tiempo de ventilación mecánica y esta puntuación (AUC=0,512). La ventilación mecánica prolongada se asoció con fármacos vasoactivos (96%;p=0,007) y una mayor gravedad de la lesión renal aguda (KDIGO=3,78%;p=0,009).

**Conclusión:**

la duración de la ventilación mecánica no se asoció con cambios en la carga de trabajo de enfermería, que se mantuvo elevada independientemente de la duración del soporte ventilatorio.

## INTRODUCTION

Invasive mechanical ventilation (IMV) is a complex ventilatory support system, widely used in the treatment of critically ill patients, often with a direct impact on survival. Its maintenance requires specialized multidisciplinary care, highlighting the central role of nursing in the care of adults with severe respiratory failure admitted to the Intensive Care Unit (ICU)^(1,2)^.

In addition to indicating clinical severity, acute respiratory failure often implies prolonged physical immobilization, associated with adverse effects such as skeletal muscle paresis, weakness, and muscle atrophy. In this context, the adoption of a structured set of collaborative care becomes essential in order to minimize such outcomes and promote functional recovery^(3)^.

Interventions related to mechanical ventilation are directly linked to advanced competencies in intensive care nursing practice^(4)^. Patient care requiring mechanical ventilation demands technical expertise in different ventilation modes, invasive devices, complication prevention strategies, and the ability to anticipate risks-essential elements for the safe management of ventilatory support^(5,6)^. Due to the multiplicity and complexity of these activities, nurses are frequently exposed to a high workload, with potential emotional and occupational repercussions, such as stress, anxiety, and reduced professional self-confidence^(7,8)^.

Given this scenario, the use of specific instruments to measure the nursing workload in intensive care is fundamental for the qualified planning of care, not limited to patients’ clinical severity, but encompassing the set of interventions effectively performed^(9)^. The Nursing Activities Score (NAS) stands out in this context for encompassing approximately 81% of nursing activities performed in the ICU and for its independence from classic disease severity scores^(9)^.

The NAS is a validated instrument in Brazil and internationally used to quantify nursing workload by measuring care activities provided in the last 24 hours, including ventilatory support, continuous monitoring, and complex procedures. The combined analysis of clinical variables, such as mortality, degree of organ failure, and acute kidney injury (AKI), allows for contextualizing patient severity and the complexity of care, favoring a more comprehensive interpretation of workload in intensive care^(10,11)^.

Despite the recognized complexity of care for patients on mechanical ventilation, objective measurement of nursing workload in this context remains limited. Recent systematic reviews point to an association between increased workload, increased clinical complexity, and worse outcomes in ICUs, highlighting the importance of using standardized instruments for safe workforce planning^(12)^. Evidence also indicates that PMV is associated with greater resource utilization, longer hospital stays, and the need for continuous intensive care^(13,14)^, factors that can substantially impact nursing care demands, especially in critical settings^(15,16)^.

Although studies have demonstrated an increased workload in mechanically ventilated patients, especially those undergoing prolonged mechanical ventilation (PMV), there are still gaps in understanding the direct association between the mechanical ventilation duration and the workload measured by specific instruments, such as the NAS. In this sense, the NAS emerges as a relevant tool to support staffing planning and the organization of care in ICUs, by systematically capturing the volume and nature of nursing interventions performed^(17,18)^.

Therefore, assessing the nursing staff’s workload in critically ill patients undergoing mechanical ventilation, differentiating between those on prolonged and non-prolonged mechanical ventilation, becomes fundamental both for the quality of care and for the efficient management of human resources in intensive care. A targeted analysis of nursing workload in this context can contribute to planning care that is proportional to patient needs, reducing care risks, and improving the quality of care provided.

## OBJECTIVE

To assess the association between IMV duration, classified as prolonged and non-prolonged, and the nursing workload in critically ill patients.

## METHODS

A prospective, quantitative cohort study, developed in accordance with the Strengthening the Reporting of Observational Studies in Epidemiology recommendations^(19)^. Data collection took place between January 2024 and January 2025 in an adult ICU of a public tertiary teaching hospital located in midwestern Brazil.

The ICU consists of 19 beds, ten for general clinical care and nine for the coronary care unit, and assists patients with high clinical and surgical complexity.

The population consisted of medical and surgical patients admitted to the ICU and undergoing mechanical ventilation. The sample was non-probabilistic, based on convenience, and comprised all eligible patients consecutively admitted to the unit during the data collection period. During data collection, we included all patients who met the inclusion criteria within the established timeframe, resulting in a final sample of 61 participants followed from the installation of mechanical ventilation until the occurrence of a clinical outcome or the end of the follow-up period.

IMV duration was considered the primary exposure variable, and was subsequently categorized into non-prolonged mechanical ventilation and PMV, according to international consensus criteria^(20)^.

### Selection criteria

Adult patients aged 18 years or older who underwent mechanical ventilation for more than 48 hours and were admitted to the ICU during the data collection period were included. Patients who died within seven days of hospitalization, those in exclusive palliative care, and those with previously diagnosed degenerative lung disease were excluded (Figure 1).

**Figure 1:**
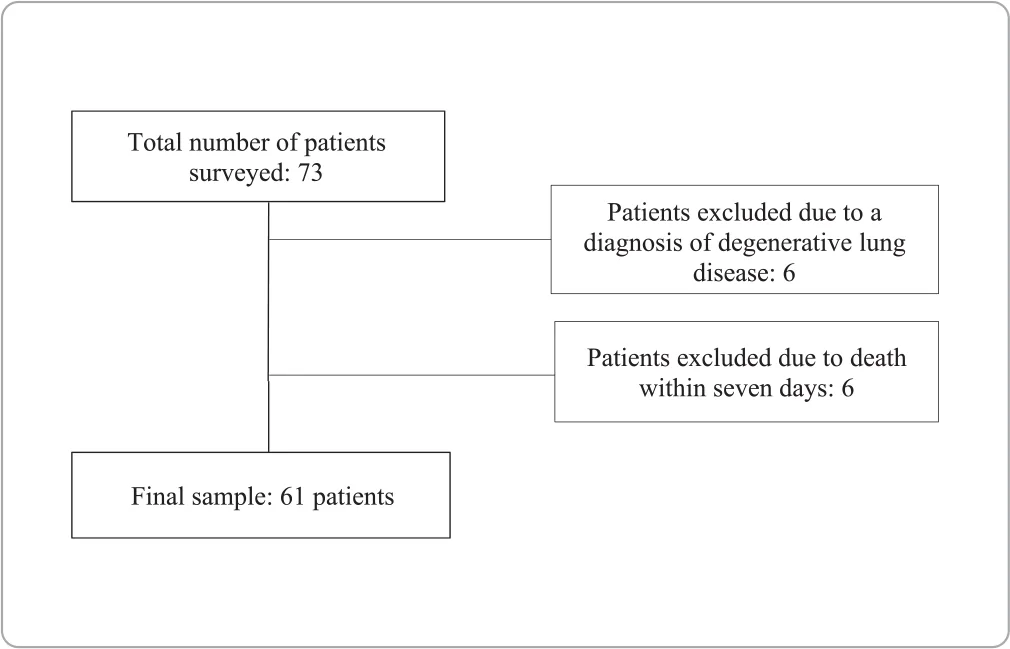
Flowchart of participant selection criteria. Brasília, Federal District, Brazil, 2025

### Data collection procedure

Data collection was prospective and systematic, carried out by two trained researchers, based on information obtained from patients’ physical and electronic medical records after they signed the Informed Consent Form.

Stage 1 - Data collection instrument construction: Initially, a structured questionnaire was developed for data collection, consisting of 38 closed-ended questions, based on scientific evidence^(4,21)^. The instrument was organized into the following sections: identification (ethnicity and sex); comorbidities (hypertension, diabetes mellitus, heart disease, and renal dysfunction); current history (use of vasoactive drugs (VADs) and date of ICU admission); ventilatory parameters (ventilatory mode, positive end-expiratory pressure (PEEP), inspired oxygen fraction, respiratory rate, date of IMV initiation, and IMV duration); laboratory tests (hydrogen ion potential, partial pressure of carbon dioxide, partial pressure of oxygen, lactate, creatinine, and serum hemoglobin); renal replacement therapy (RRT); and clinical outcome (discharge, transfer to another unit, transfer to another hospital, death, and palliative care).

Nursing workload was measured daily using the NAS, a validated instrument that assesses, over a 24-hour period, the care demands of critically ill patients based on activities of direct care, monitoring, therapeutic interventions, ventilatory support, mobilization, and administrative tasks. The NAS score expresses the proportion of a nursing professional’s time dedicated to patient care.

Patients’ clinical severity and mortality risk were assessed using the Simplified Acute Physiology Score III (SAPS III) and the Sequential Organ Failure Assessment (SOFA) scores.

Stage 2 - Participant identification, recruitment, and follow-up: participant identification occurred through daily monitoring of admissions to the unit, consecutively including all eligible patients. Participants were prospectively followed for up to 30 days, from ICU admission until the occurrence of the clinical outcome or the end of follow-up.

Data collection was performed prospectively using a structured instrument, with information extracted from physical and electronic medical records and care records. The variables and instruments used were selected to directly address the study’s objective, allowing for the assessment of the relationship between IMV time, nursing workload, clinical severity, and patient outcomes.

Stage 3 - Clinical outcome assessment: at the end of follow-up, participants’ clinical outcomes were recorded, categorized as ICU discharge, transfer to another unit, inter-hospital transfer, or death.

### Study variables

PMV was defined based on the consensus of the National Association for Medical Direction of Respiratory Care, which defines IMV for a period equal to or greater than 21 consecutive days, for at least six hours daily^(20)^.

The nursing staff’s workload was measured using the NAS, a tool that considers ventilatory, cardiovascular, renal, neurological, and metabolic support, basic activities, and interventions performed in patient care. It is applied once a day, reflecting the activities performed in the previous 24 hours. The score is expressed as a percentage, ranging from 0% to 176.8%, with 100% corresponding to the need for a full-time nursing professional for patients^(9,22)^.

Clinical severity was assessed using the SOFA score, used in ICUs to measure the degree of organ failure, and should be applied upon admission and then daily^(23,24)^. It assesses six systems: cardiovascular, hepatic, respiratory, coagulation, renal, and neurological. Each receives a score from 0 to 4, with higher scores indicating greater dysfunction^(24)^. The SAPS III, composed of 20 parameters, is used upon ICU admission as a prognostic indicator of mortality^(25,26)^. The parameters are grouped into three categories: patients’ pre-existing conditions, circumstances of admission, and physiological dysfunction^(26)^.

The inclusion of clinical severity indicators in the data collection instrument is justified by their relevance in characterizing the complexity of care and measuring care demands that influence both the patient’s clinical evolution and the nursing staff’s workload. Recent evidence indicates that high clinical severity scores are associated with a greater need for nursing interventions, increased care time demands, and higher NAS, reinforcing the relevance of integrating them into the data collection instrument and the study analysis^(11,12)^. AKI was defined according to the creatinine criteria of the Kidney Disease: Improving Global Outcomes (KDIGO) study^(27)^. The severity of AKI is stratified as follows: KDIGO 1 (risk for kidney injury), when there is an increase in serum creatinine (sCr) of 0.3 mg/dL in 48 hours or an increase of 1.5 times or 1.9 times the baseline value in seven days; KDIGO 2 (kidney injury), when the increase in sCr is 2.0 to 2.9 times the baseline value; and KDIGO 3 (kidney failure), when the increase in sCr is 3.0 times the baseline value or when RRT is required^(27)^. The lowest creatinine level recorded between seven and 365 days prior to ICU admission was considered as baseline^(3)^.

Acute kidney disease (AKD) was characterized by the persistence of AKI for a period between seven and 90 days after the triggering event^(3)^. Recovery of renal function was calculated in patients with AKI using the ratio of sCr at the time of assessment to baseline sCr. There are different levels of recovery, such as full recovery of renal function, which occurs when creatinine returns to the baseline sCr value^(3)^.

### Data processing

In statistical analysis, descriptive measures such as mean, median, standard deviation, interquartile range, absolute frequency, and percentages were calculated to describe the characteristics of variables. The chi-square test was used to investigate the association between different categorical variables. Fisher’s exact test was applied when the sample size was small, allowing the assessment of the association between two categorical variables when the conditions for the applicability of the chi-square test were not met.

In this study, normality was not observed in the data. Therefore, the Wilcoxon-Mann-Whitney test was used to compare the medians of two samples (dependent or independent) in situations where the data did not meet the assumptions of normal distribution and homogeneity of variances. In logistic regression, the coefficients of independent variables were interpreted in terms of Odds Ratio, indicating how much the chances of the event change with a one-unit increase in the variable. To measure the predictive capacity of the selected NAS variables in relation to mechanical ventilation time, the Receiver Operating Characteristic (ROC) curve was constructed.

In the present study, all statistical analyses were performed using the R programming environment (version 4.3.2) (R Core Team, 2023), and the significance level adopted was 5%.

### Ethical aspects

The study was conducted in accordance with national and international ethical guidelines. It was approved by the *Universidade de Brasília* Research Ethics Committee, School of Health Sciences, whose opinion is attached to this submission. Informed consent was obtained by the researchers from participants or, if they were unable to consent, from their legal representatives, prior to inclusion in the study.

## RESULTS

Among the 61 patients monitored, the mean age was 56 years, with a predominance of individuals over 60 years of age in the group with PMV (> 21 days). The mixed-race ethnicity was the most frequent in all groups, regardless of ventilation time. A predominance of males was observed in the total sample, especially among patients on mechanical ventilation. The most prevalent comorbidities were diseases of the circulatory system (63%) and endocrine system (52%), occurring more frequently in the group undergoing PMV.

The use of VADs was significantly more frequent in the group with mechanical ventilation (p = 0.007), with a median of 17 days, highlighting the predominance of vasopressor drugs (96%) compared to the group with ventilation for less than 21 days (82%). RRT was also more common among patients on PMV (70%). IMV duration showed a statistically significant difference, with a median of 30 days in the PMV group and 13 days in the non-prolonged mechanical ventilation group (p < 0.001).

The median SAPS III score was higher in the group with PMV, with a value of 74. Nursing workload, measured by the NAS, showed high values throughout the sample, with a median of 112% (interquartile range: 106-119), indicating a care demand exceeding the full-time availability of a nursing professional per patient in 24 hours. In the comparison between groups, no statistically significant differences were observed between the NAS scores in the groups with mechanical ventilation time less than or greater than 21 days. The in-hospital mortality rate was 41% in the group on PMV, compared to 50% in the group with non-prolonged mechanical ventilation. Severe AKI was more frequent in the PMV group (78%) compared to the group without PMV (71%). Both AKI and AKD occurred more frequently in the PMV group (96% and 58%, respectively). Recovery of renal function was significantly greater among patients with non-prolonged ventilation (56%) compared to those with PMV (27%; p = 0.034) (Table 1).

**Table 1:** Demographic and clinical characterization according to the time (days) of invasive mechanical ventilation. Brasília, Federal District, Brazil, 2025

		Invasive mechanical ventilation		
**Characteristics**	**Total, N = 61**	**≤21, N = 34**	**>21, N = 27**	***p* value**
Age (years)				0.079^1^
Mean (standard deviation)	56 (16)	53 (18)	60 (14)	
Median [IQR]	58 [44. 69]	53 [42. 64]	63 [53. 72]	
Male, n/N (%)	31/61 (51%)	15/34 (44%)	16/27 (59%)	0.240^2^
Ethnicity, n/N (%)				0.257^3^
Black	2/61 (3.3%)	1/34 (2.9%)	1/27 (3.7%)	
White	12/61 (20%)	4/34 (12%)	8/27 (30%)	
Indigenous	1/61 (1.6%)	1/34 (2.9%)	0/27 (0%)	
Mixed-race	45/61 (74%)	27/34 (79%)	18/27 (67%)	
Asian	1/61 (1.6%)	1/34 (2.9%)	0/27 (0%)	
Comorbidities				
Cardiopathy, n/N (%)	21/61 (34%)	13/34 (38%)	8/27 (30%)	0.482^2^
Endocrine, n/N (%)	26/61 (43%)	12/34 (35%)	14/27 (52%)	0.194^2^
Circulatory, n/N (%)	31/61 (51%)	14/34 (41%)	17/27 (63%)	0.091^2^
Autoimmune, n/N (%)	10/61 (16%)	4/34 (12%)	6/27 (22%)	0.315^3^
Musculoskeletal, n/N (%)	5/61 (8.2%)	3/34 (8.8%)	2/27 (7.4%)	>0.999^3^
Infections, n/N (%)	10/61 (16%)	3/34 (8.8%)	7/27 (26%)	0.092^3^
Oncohemato, n/N (%)	27/61 (44%)	17/34 (50%)	10/27 (37%)	0.311^2^
Vasopressor, n/N (%)	54/61 (89%)	28/34 (82%)	26/27 (96%)	0.121^3^
Vasodilator, n/N (%)	4/61 (6.6%)	4/34 (12%)	0/27 (0%)	0.122^3^
Inotropic, n/N (%)	12/61 (20%)	7/34 (21%)	5/27 (19%)	0.840^2^
Total VAD days				0.007^1^
Mean (standard deviation)	14 (10)	12 (10)	17 (9)	
Median [IQR]	12 [8. 18]	9 [6. 15]	17 [11. 23]	
SAPS III				0.090^1^
Mean (standard deviation)	70 (16)	67 (18)	74 (11)	
Median [IQR]	73 [59. 80]	68 [52. 77]	74 [65. 82]	
NAS (%)				0.880^1^
Mean (standard deviation)	113 (12)	112 (14)	113 (11)	
Median [IQR]	112 [106. 119]	111.2 [105. 118]	111.8 [107. 119]	
RRT, n/N (%)	38/61 (62%)	19/34 (56%)	19/27 (70%)	0.246^2^
Total RRT days				0.009^1^
Mean (standard deviation)	13 (9)	8 (6)	17 (10)	
Median [IQR]	12 [5. 18]	7 [5. 11]	15 [13. 23]	
Time on IMV (days)				<0.001^1^
Mean (standard deviation)	21 (13)	13 (5)	32 (11)	
Median [IQR]	20 [11. 28]	13 [8. 17]	30 [24. 37]	
Baseline creatinine				0.940^1^
Mean (standard deviation)	1.33 (1.23)	1.46 (1.48)	1.15 (0.78)	
Median [IQR]	0.90 [0.60. 1.40]	0.95 [0.60. 1.40]	0.85 [0.70. 1.38]	
Outcome, n/N (%)				0.179^3^
Discharge	1/61 (1.6%)	1/34 (2.9%)	0/27 (0%)	
Entered palliative care	12/61 (20%)	4/34 (12%)	8/27 (30%)	
Death	28/61 (46%)	17/34 (50%)	11/27 (41%)	
Transfer to another hospital	6/61 (9.8%)	2/34 (5.9%)	4/27 (15%)	
Transfer to another sector	14/61 (23%)	10/34 (29%)	4/27 (15%)	
In-hospital death, n/N (%)	28/61 (46%)	17/34 (50%)	11/27 (41%)	0.471^2^
KDIGO, n/N (%)				0.197^3^
0	8/61 (13%)	7/34 (21%)	1/27 (3.7%)	
1	5/61 (8.2%)	2/34 (5.9%)	3/27 (11%)	
2	3/61 (4.9%)	1/34 (2.9%)	2/27 (7.4%)	
3	45/61 (74%)	24/34 (71%)	21/27 (78%)	
AKI, n/N (%)	53/61 (87%)	27/34 (79%)	26/27 (96%)	0.066^3^
AKD, n/N (%)	21/39 (54%)	7/15 (47%)	14/24 (58%)	0.477^2^
Recovery of kidney function, n/N (%)	22/53 (42%)	15/27 (56%)	7/26 (27%)	0.034^2^

Legend:

1 Wilcoxon rank-sum test;

2 Chi-square test of independence;

3 Fisher’s exact test; IQR - interquartile range; VAD - vasoactive drug; RRT - renal replacement therapy; IMV - invasive mechanical ventilation; KDIGO - Kidney Disease: Improving Global Outcomes; AKI - acute kidney injury; AKD - acute kidney disease; NAS - Nursing Activities Score.

Assisted-controlled ventilation was the most frequently used mode in both groups. Ventilatory parameters, as well as arterial blood gas laboratory data, did not show significant differences between the groups (Table 2).

**Table 2:** Comparison of laboratory and ventilatory variables and invasive mechanical ventilation duration. Brasília, Federal District, Brazil, 2025

		Invasive mechanical ventilation		
**Characteristics**	**Total, N = 61**	**≤21, N = 34**	**>21, N = 27**	***p* value**
Mean PEEP (cm/H_2_O)				0.217^1^
Mean (standard deviation)	6.81 (1.05)	6.99 (1.20)	6.59 (0.78)	
Median [IQR]	6.62 [6.18. 7.50]	6.72 [6.23. 7.59]	6.53 [6.12. 6.88]	
Mean FiO_2_ (%)				0.289^1^
Mean (standard deviation)	33 (12)	34 (12)	31 (12)	
Median [IQR]	29 [26. 33]	30 [26. 38]	29 [26. 31]	
Ventilatory mode, n/N (%)				0.283^2^
A/C	53/61 (87%)	28/34 (82%)	25/27 (93%)	
Spontaneous	8/61 (13%)	6/34 (18%)	2/27 (7.4%)	
Mean pH				0.380^1^
Mean (standard deviation)	7.41 (0.05)	7.40 (0.06)	7.41 (0.04)	
Median [IQR]	7.41 [7.38. 7.43]	7.40 [7.36. 7.43]	7.41 [7.39. 7.43]	
Mean PaO_2_ (mmHg)				0.149^1^
Mean (standard deviation)	86 (17)	90 (20)	82 (10)	
Median [IQR]	83 [76. 92]	83 [80. 93]	81 [75. 88]	
Mean PaO_2_ (mmHg)				0.493^1^
Mean (standard deviation)	37 (8)	37 (7)	38 (8)	
Median [IQR]	36 [32. 40]	36 [32. 40]	36 [33. 40]	
Mean bicarbonate (mEq/L)				0.880^1^
Mean (standard deviation)	23.15 (3.82)	23.41 (4.89)	22.82 (1.77)	
Median [IQR]	22.84 [21.55. 24.43]	22.38 [21.12. 25.01]	23.42 [21.92. 24.15]	
Mean lactate (mg/dL)				0.902^1^
Mean (standard deviation)	20 (19)	24 (24)	16 (5)	
Median [IQR]	16 [11. 19]	15 [10. 28]	16 [12. 18]	
Mean hemoglobin (g/dL)				0.540^1^
Mean (standard deviation)	8.40 (1.50)	8.45 (1.64)	8.34 (1.34)	
Median [IQR]	8.08 [7.60. 8.86]	8.24 [7.63. 9.13]	8.00 [7.56. 8.60]	

Legend:

1 Wilcoxon rank-sum test;

2 Teste exato de Fisher Model; PEEP - positive end-expiratory pressure; IQR - interquartile range; FiO_2_ - fraction of inspired oxygen; SOFA - Sequential Organ Failure Assessment; A/C - assisted-controlled; pH - potential of hydrogen; PaO_2_ - partial pressure of oxygen; PaCO_2_ - partial pressure of carbon dioxide.

Multivariate analysis indicated that VAD use duration was an independent risk factor for the occurrence of PMV (p=0.050) (Table 3).

**Table 3:** Multivariate analysis of clinical variables and mechanical ventilation time. Brasília, Federal District, Brazil, 2025

	Univariate		Adjusted		Multivariate	
**Characteristics**	**OR (95%CI)**	***p* value**	**OR (95%CI)**	***p* value**	**OR (95%CI)**	***p* value**
Age (years)	1.02 (0.99-1.06)	0.169	1.00 (0.95-1.05)	0.898		
Endocrine	1.64 (0.56-4.88)	0.369	2.18 (0.46-10.6)	0.320		
Circulatory	1.97 (0.68-5.92)	0.217	1.66 (0.37-7.80)	0.509		
Infections	3.19 (0.78-16.3)	0.123	4.32 (0.71-34.6)	0.131		
Total VAD days	1.07 (1.01-1.15)	0.050	1.06 (0.99-1.16)	0.124	1.07 (1.01-1.15)	0.050
SAPS III	1.02 (0.98-1.06)	0.285	1.02 (0.96-1.10)	0.451		
SOFA at admission	1.08 (0.90-1.30)	0.415	0.96 (0.71-1.30)	0.784		
KDIGO	1.30 (0.75-2.45)	0.366	1.20 (0.30-4.51)	0.780		
AKI	4.00 (0.54-81.2)	0.229	2.16 (0.04-194)	0.710		
Mean PaO_2_	0.97 (0.92-1.00)	0.134	0.95 (0.88-1.00)	0.055		

*Legend: OR - Odds Ratio; CI - Confidence Interval; VAD - vasoactive drug; SAPS III - Simplified Acute Physiology Score III; SOFA - Sequential Organ Failure Assessment; KDIGO - Kidney Disease: Improving Global Outcomes; AKI - acute kidney injury; PaO_2_ - partial pressure of oxygen.*

Although patients undergoing PMV presented greater clinical complexity, characterized by increased use of VADs, need for respiratory therapy, and higher frequency of AKI, these differences were not reflected in an increased nursing workload, as demonstrated by the maintenance of similar NAS scores between the groups (Figure 2).

**Figure 2:**
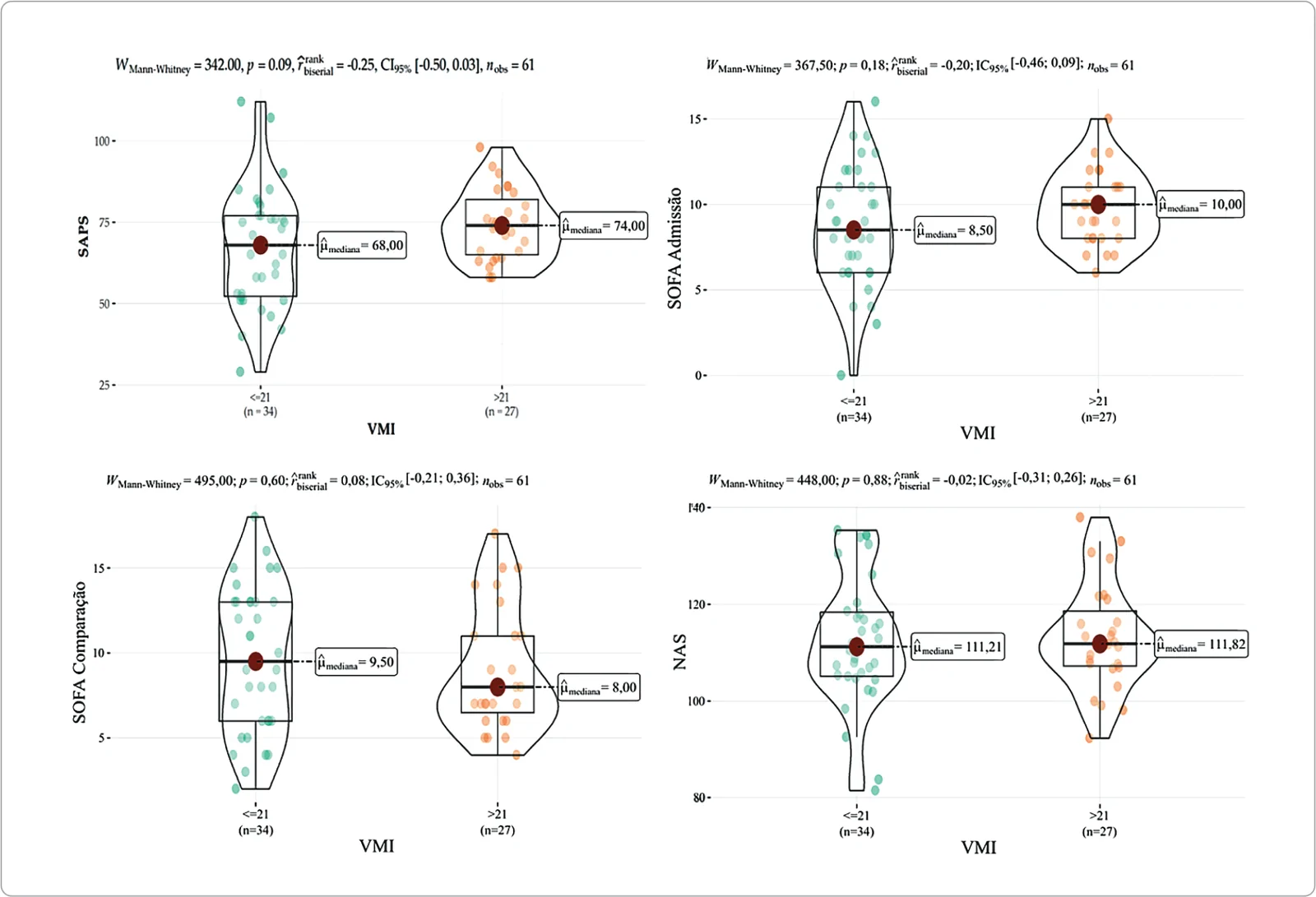
Comparison of Simplified Acute Physiology Score, Sequential Organ Failure Assessment, and Nursing Activities Score with invasive mechanical ventilation time in days. Brasília, Federal District, Brazil, 2025

The clinical severity of patients, assessed by SAPS III and SOFA, did not show significant variations in relation to IMV duration. It was observed that, at admission, there was an increase in SAPS III and SOFA scores in the group on mechanical ventilation for a period exceeding 21 days, although, during the same period, the NAS did not show a difference between the groups. The reduction in SOFA scores was identified in the outcome, indicating a reduction in patient severity over the ventilation period (Figure 2).

No significant association was identified between IMV duration and the nursing staff’s workload, as measured by the NAS, with an area under the curve (AUC) of 0.512, indicating that IMV duration did not influence this outcome.

## DISCUSSION

In the present study, no significant association was observed between IMV duration and the nursing staff’s workload in critically ill patients, as measured by the NAS. Although the median NAS scores were above 100% in both groups, with IMV less than 21 days and with PMV, indicating a high overall care demand, this difference was not statistically significant. Analysis of the ROC curve supported this finding, with an AUC of 0.512, demonstrating low discriminatory power between mechanical ventilation time and nursing staff workload. These results indicate that IMV duration, in isolation, was not a determining factor in the increase in workload measured by the NAS.

It is noteworthy that patients undergoing PMV presented greater clinical complexity, evidenced by greater use of VADs, a greater need for RRT, and a higher frequency of AKI. However, this greater clinical severity was not reflected in an increased nursing workload between the groups. This finding reinforces that the NAS measures the volume and nature of nursing interventions performed over a 24-hour period, and is not determined exclusively by clinical severity or ventilatory support duration, but rather by the set of care effectively provided to the critically ill patient^(28)^.

Evidence indicates that a NAS score of 100 corresponds to the equivalent demand of one nursing professional per patient; however, studies show that scores above 61 are associated with increased hospital mortality^(29)^. In the present study, a NAS score greater than 100 in both groups reflects a high care burden on the nursing staff, regardless of the mechanical ventilation duration. Furthermore, almost half of patients died in the hospital, highlighting the clinical severity of the sample, supported by the SAPS III and SOFA prognostic scores, especially among patients on PMV.

Patients’ clinical severity, as expressed by prognostic scores, suggests a greater demand for nursing care due to the complexity of the interventions performed in intensive care, such as a greater need for ventilatory support, use of VADs, diuretics, and continuous monitoring. These interventions, fundamental for clinical stabilization, increase the complexity of care and require constant vigilance from the nursing staff^(28)^. However, even in the face of these demands, the NAS remained similar between the groups, suggesting that such care is part of the routine care for critically ill patients on mechanical ventilation, regardless of the length of time they remain on this support^(30,31)^.

Despite advances in therapeutic interventions, mortality among patients undergoing mechanical ventilation remains high, with longer ventilatory support duration associated with worse clinical outcomes^(29)^. The ability to predict IMV duration early can contribute to the planning of proactive interventions, the optimization of care, and cost reduction, as well as providing support to patients and their families^(32)^. However, the results of the present study indicate that the workload of the nursing staff remains high even in patients with shorter ventilation times, reinforcing that the burden of care is not restricted to prolonged ventilation.

A retrospective cohort study conducted in Uruguay identified a high demand for mechanical ventilation in ICUs, with 17% of patients requiring PMV^(33)^. In the present investigation, 44% of patients on IMV progressed to PMV, a higher value than described in the literature. These patients, frequently classified as chronically critically ill, present persistent inflammation and greater dependence on supportive therapies such as RRT and VADs^(34,35)^. In this scenario, the present study confirmed a greater need for prolonged RRT in the group with PMV, reflecting greater clinical severity, but without a significant additional association in the nursing staff’s workload.

Older patients and those with multiple comorbidities at admission are more predisposed to PMV (for more than 21 days)^(34)^. A systematic review conducted in Pakistan also demonstrated that the presence of multimorbidity is associated with the development of complications, such as AKI, and progression to PMV^(36)^. In the present study, a high frequency of severe AKI was observed among critically ill patients; however, the recovery of renal function was significant, especially among those with shorter ventilation times, suggesting appropriate clinical management.

IMV is an essential ventilatory support for restoring gas exchange, but it can induce or worsen lung injuries and trigger inflammatory processes and oxidative stress^(37)^. Despite this, in this study, no significant differences were observed in blood gas parameters between the groups throughout the follow-up, indicating similar metabolic stability regardless of ventilation time. From a nursing perspective, this finding reinforces that the workload stems primarily from continuous care needs, such as monitoring, adjustment of ventilatory parameters, and general care, and not exclusively from measurable physiological changes^(38)^.

In the care of patients on IMV, nursing plays a central role in the implementation of care protocols, continuous assessment of comfort, agitation, and sedation levels. These interventions require frequent observation and clinical decision-making, directly impacting workload and contributing to increased sedation levels, even in patients with shorter ventilation times^(39,40)^.

The use of VADs was high in the studied sample, being observed in 89% of patients. A study carried out in Australia identified 84% of critically ill patients using VADs who used vasopressors^(41)^, a finding consistent with the present study. The administration and titration of these drugs require continuous monitoring, frequent record-keeping, and constant clinical assessments, which contributes to an increased workload for the nursing staff^(42)^.

Several medications used in the ICU have nephrotoxic potential and are associated with the development of AKI^(43)^. In the present study, AKI was observed in more than half of patients, regardless of mechanical ventilation duration. The management of these patients requires rigorous monitoring, fluid control, and frequent laboratory assessment, activities that increase the demand for care from the nursing staff.

Although evidence suggests an association between AKI and longer mechanical ventilation time^(44,45)^, the present study did not identify a statistically significant association between these variables, although a trend toward longer IMV time was observed among patients with AKI. Furthermore, a significant correlation was observed between the total number of days on RRT and the time on IMV, reflecting clinical severity and the presence of multisystem dysfunctions.

Recovery of renal function was significantly greater among patients with shorter mechanical ventilation time (p=0.034), a finding similar to that observed in a historical, retrospective, and quantitative cohort study conducted in Brazil that associates early weaning from mechanical ventilation with improved renal function^(46)^. These results reinforce the importance of proper management of ventilatory support, with a potential indirect association with nursing workload throughout hospitalization.

The high mortality rate observed in the study is consistent with the literature, which associates greater clinical severity and multiple organ dysfunction with worse outcomes in patients undergoing IMV^(47)^. The SAPS III and SOFA scores proved to be useful tools in prognostic assessment, with higher values among patients in PMV, supporting findings from other studies^(46,47)^.

The results of this research show that the workload of the nursing staff in critically ill patients undergoing mechanical ventilation is high, regardless of the mechanical ventilation duration, reinforcing the need for adequate team sizing and care planning. Accurate measurement of workload through the NAS allows for adjusting the nurse-to-patient ratio to the effective hours of care, contributing to the quality of care, patient safety, and reduction of professional overload^(48,49)^.

### Study limitations

The limitations of this study include the single-center design and small sample size, factors that may restrict the generalizability of results to other care settings. Furthermore, although the NAS encompasses nursing activities performed over a 24-hour period, its application was conducted once a day, which may not reflect intra-hourly variations in workload. The presence of incomplete records in electronic medical records may also have influenced the accuracy of NAS analysis.

### Contributions to nursing, health, or public policy

This study contributes to expanding knowledge about the relationship between IMV and the nursing staff’s workload in ICUs, by analyzing in an integrated way ventilatory support duration and the measurement of care through the NAS. By showing that the workload remains high regardless of the mechanical ventilation duration, the findings provide support for care planning, appropriate staffing of the nursing team, and improvement of care for critically ill patients undergoing PMV and non-prolonged mechanical ventilation.

## CONCLUSIONS

Mechanical ventilation duration was not associated with changes in nursing workload, which remained high regardless of the ventilatory support duration. Mechanical ventilation was associated with greater clinical severity, reflected by a high incidence of AKI and high prognostic scores. These findings demonstrate that the burden on nursing staff for critically ill patients undergoing mechanical ventilation stems primarily from the clinical complexity and multiple interventions required, and not exclusively from the length of time on mechanical ventilation.

## Data Availability

The available research data is in the body of the article.
