# Supplementary material for: Nursing Activities Score in critically ill patients on invasive mechanical ventilation
Source: Rev Bras Enferm. 2026 Oct 19;79:e20250390. doi: 10.1590/0034-7167-2025-0390 (PMC13679590; doi:10.1590/0034-7167-2025-0390)
Supplement: Translation

# Translation: *Nursing Activities Score* em pacientes críticos em ventilação mecânica invasiva

**DOI:** 10.1590/0034-7167-2025-0390pt

**Authors:** Larissa Moreira Bianchi, Tayse Tâmara da Paixão Duarte, Michelle Zampieri Ipolito, Kamilla Grasielle Nunes da Silva, Walterlânia Silva Santos, Marcia Cristina da Silva Magro

**Affiliations:** I Universidade de Brasília, Faculdade de Ciências e Tecnologia em Saúde. Brasília, Distrito Federal, Brasil; II Universidade de Brasília, Hospital Universitário de Brasília. Brasília, Distrito Federal, Brasil

**Keywords:** Respiração Artificial, Carga de Trabalho, Enfermagem, Escores de Disfunção Orgânica, Cuidados Críticos.

## Abstract

**Objetivo:**

avaliar a associação entre o tempo de ventilação mecânica invasiva, classificada como prolongada e não prolongada, e a carga de trabalho da enfermagem em pacientes críticos.

**Método:**

estudo de coorte prospectivo com pacientes adultos em ventilação mecânica por até 21 dias ou mais. Carga de trabalho da enfermagem foi mensurada pelo Nursing Activities Score (NAS). Coletaram-se dados clínicos por questionário estruturado. Adotou-se nível de significância de 5%.

**Resultados:**

considerando 61 pacientes, não houve diferença significativa na carga de trabalho da enfermagem entre ventilação mecânica prolongada e não prolongada segundo NAS [111,2(105,118)*vs.*111,8(107,119); p=0,880], nem associação entre tempo de ventilação mecânica e esse escore (AUC=0,512). Ventilação mecânica prolongada se associou com drogas vasoativas (96%; p=0,007;) e maior gravidade da injúria renal aguda (KDIGO=3,78%; p=0,009).

**Conclusão:**

o tempo de ventilação mecânica não se associou à variação da carga de trabalho da enfermagem, que permaneceu elevada independentemente da duração do suporte ventilatório.

## INTRODUÇÃO

A ventilação mecânica invasiva (VMI) constitui um suporte ventilatório complexo, amplamente utilizado no tratamento de pacientes críticos, frequentemente com repercussão direta na sobrevida. Sua manutenção demanda atuação multiprofissional especializada, destacando-se o papel central da enfermagem no cuidado de adultos com insuficiência respiratória grave internados em Unidade de Terapia Intensiva (UTI)^(1,2)^.

Além de indicar gravidade clínica, a insuficiência respiratória aguda frequentemente implica a imobilização física prolongada, associada a efeitos adversos como paresia de músculos esqueléticos, fraqueza e atrofia muscular. Nesse contexto, torna-se imprescindível a adoção de um conjunto estruturado de cuidados colaborativos com o objetivo de minimizar tais desfechos e favorecer a recuperação funcional^(3)^.

As intervenções relacionadas à VMI estão diretamente vinculadas às competências avançadas da prática de enfermagem em terapia intensiva^(4)^. O cuidado ao paciente em VMI exige domínio técnico sobre diferentes modos ventilatórios, dispositivos invasivos, estratégias de prevenção de complicações e capacidade de antecipação de riscos, elementos essenciais para manejo seguro do suporte ventilatório^(5,6)^. Em razão da multiplicidade e da complexidade dessas atividades, os enfermeiros frequentemente estão expostos a uma elevada carga de trabalho, com potenciais repercussões emocionais e ocupacionais, como estresse, ansiedade e redução da autoconfiança profissional^(7,8)^.

Diante desse cenário, o uso de instrumentos específicos para mensuração da carga de trabalho da enfermagem em cuidados intensivos se mostra fundamental para o planejamento qualificado da assistência, não se limitando à gravidade clínica do paciente, mas contemplando o conjunto de intervenções efetivamente realizadas^(9)^. O *Nursing Activities Score* (NAS) se destaca nesse contexto por abranger aproximadamente 81% das atividades de enfermagem desenvolvidas em UTI e por apresentar independência em relação aos escores clássicos de gravidade da doença^(9)^.

O NAS é um instrumento validado no Brasil e internacionalmente utilizado para quantificar a carga de trabalho da enfermagem a partir da mensuração das atividades de cuidado prestadas nas últimas 24 horas, incluindo suporte ventilatório, monitorização contínua e procedimentos complexos. A análise conjunta de variáveis clínicas, como mortalidade, grau de falência orgânica e injúria renal aguda (IRA), permite contextualizar a gravidade dos pacientes e a complexidade da assistência, favorecendo uma interpretação mais abrangente da sobrecarga de trabalho em terapia intensiva^(10,11)^.

Apesar da reconhecida complexidade assistencial dos pacientes em VMI, a mensuração objetiva da carga de trabalho de enfermagem nesse contexto ainda é limitada. Revisões sistemáticas recentes apontam associação entre maior carga de trabalho, aumento da complexidade clínica e piores desfechos em UTIs, ressaltando a importância do uso de instrumentos padronizados para o planejamento seguro da força de trabalho^(12)^. Evidências também indicam que a VMP está associada a uma maior utilização de recursos, um maior tempo de internação e à necessidade de cuidados intensivos contínuos^(13,14)^, fatores que podem impactar substancialmente a demanda assistencial da enfermagem, especialmente em cenários críticos^(15,16)^.

Embora estudos tenham demonstrado aumento da carga de trabalho em pacientes ventilados mecanicamente, especialmente naqueles submetidos à VMI prolongada, ainda existem lacunas quanto à compreensão da associação direta entre o tempo de ventilação mecânica e a carga de trabalho mensurada por instrumentos específicos, como o NAS. Nesse sentido, o NAS surge como ferramenta relevante para subsidiar o dimensionamento de pessoal e a organização do cuidado em UTIs, por captar de forma sistematizada o volume e a natureza das intervenções de enfermagem realizadas^(17,18)^.

Diante disso, avaliar a carga de trabalho da equipe de enfermagem em pacientes críticos submetidos à VMI, diferenciando aqueles em VMP e não prolongada, torna-se fundamental tanto para a qualidade da assistência quanto para a gestão eficiente dos recursos humanos em terapia intensiva. Uma análise direcionada da carga de trabalho da enfermagem nesse contexto pode contribuir para o planejamento do cuidado proporcional às demandas do paciente, reduzir riscos assistenciais e aprimorar a qualidade do cuidado prestado.

## OBJETIVO

Avaliar a associação entre o tempo de VMI, classificada como prolongada e não prolongada, e a carga de trabalho da enfermagem em pacientes críticos.

## MÉTODOS

Estudo de coorte, prospectivo, de abordagem quantitativa, desenvolvido conforme as recomendações do *STrengthening the Reporting of OBservational studies in Epidemiology* ^(19)^. A coleta de dados foi realizada no período de janeiro de 2024 a janeiro de 2025 em uma UTI adulto de um hospital público de ensino terciário localizado na região Centro-Oeste do Brasil.

A UTI é constituída de 19 leitos, sendo dez destinados à clínica geral e nove à unidade coronariana, e atende pacientes de alta complexidade clínica e cirúrgica.

A população foi constituída por pacientes clínicos e cirúrgicos internados na UTI e submetidos à VMI. A amostra foi não probabilística e de conveniência, composta por todos os pacientes elegíveis internados de forma consecutiva na unidade durante o período de coleta. Durante a coleta de dados, incluímos a totalidade dos pacientes que atenderam aos critérios de inclusão no intervalo estabelecido, resultando em uma amostra final de 61 participantes acompanhados desde a instalação da VMI até a ocorrência de desfecho clínico ou o final do período de seguimento.

O tempo de VMI foi considerado a variável de exposição principal, sendo posteriormente categorizado em ventilação mecânica não prolongada e VMP, conforme critério de consenso internacional^(20)^.

### Critérios de seleção

Foram incluídos pacientes adultos, com idade igual ou superior a 18 anos, submetidos à VMI por período superior a 48 horas e internados na UTI durante o período de coleta de dados. Excluíram-se os pacientes que evoluíram para óbito em período inferior a sete dias de internação, em cuidados paliativos exclusivos e que apresentavam doença pulmonar degenerativa previamente diagnosticada (Figura 1).

**Figura 1:** Fluxograma do critério de seleção dos participantes. Brasília, Distrito Federal, Brasil, 2025

### Procedimento de coleta de dados

A coleta de dados ocorreu de forma prospectiva e sistematizada, realizada por dois pesquisadores treinados, a partir de informações obtidas nos prontuários físicos e eletrônicos dos pacientes, após a assinatura do Termo de Consentimento Livre e Esclarecido.

Etapa 1 - Construção do instrumento de coleta de dados: inicialmente, foi desenvolvido um questionário estruturado para coleta de dados, composto por 38 questões fechadas, elaborado com base em evidências científicas^(4,21)^. O instrumento foi organizado nas seguintes seções: identificação (etnia e sexo); comorbidades (hipertensão arterial, diabetes mellitus, cardiopatias e disfunção renal); história atual (uso de drogas vasoativas (DVAs) e data de internação na UTI); parâmetros ventilatórios (modo ventilatório, pressão positiva no final da expiração (PEEP), fração inspirada de oxigênio, frequência respiratória, data de instalação da VMI e tempo de VMI); exames laboratoriais (potencial hidrogeniônico, pressão parcial de gás carbônico, pressão parcial de oxigênio, lactato, creatinina e hemoglobina sérica); terapia de substituição renal (TSR); e desfecho clínico (alta, transferência para outro setor, transferência para outro hospital, óbito e cuidados paliativos).

A carga de trabalho da enfermagem foi mensurada diariamente por meio do NAS, instrumento validado que avalia, em um período de 24 horas, a demanda assistencial do paciente crítico com base nas atividades de cuidado direto, monitorização, intervenções terapêuticas, suporte ventilatório, mobilização e atividades administrativas. A pontuação do NAS expressa a proporção do tempo de um profissional de enfermagem dedicado à assistência ao paciente.

A gravidade clínica e o risco de mortalidade dos pacientes foram avaliados pelos *Simplified Acute Physiology Score III* (SAPS III) e pelo *Sequential Organ Failure Assessment* (SOFA).

Etapa 2 - Identificação, recrutamento e seguimento dos participantes: a identificação dos participantes ocorreu por meio do acompanhamento diário das admissões na unidade, incluindo-se, de forma consecutiva, todos os pacientes elegíveis. Os participantes foram acompanhados prospectivamente por até 30 dias, desde a admissão na UTI até a ocorrência do desfecho clínico ou encerramento do seguimento.

A coleta de dados foi realizada prospectivamente por meio de instrumento estruturado, com informações extraídas do prontuário físico e eletrônico, bem como de registros assistenciais. As variáveis e instrumentos utilizados foram selecionados para responder diretamente ao objetivo do estudo, permitindo avaliar a relação entre tempo de VMI, carga de trabalho da enfermagem, gravidade clínica e desfechos dos pacientes.

Etapa 3 - Avaliação dos desfechos clínicos: ao final do acompanhamento, foram registrados os desfechos clínicos dos participantes, categorizados como alta da UTI, transferência para outro setor, transferência inter-hospitalar ou óbito.

### Variáveis do estudo

VMP foi definida com base no consenso da National Association for Medical Direction of Respiratory Care, que define VMI por período igual ou superior a 21 dias consecutivos por pelo menos seis horas diárias^(20)^.

A carga de trabalho da equipe de enfermagem foi mensurada por meio do NAS, ferramenta que considera suporte ventilatório, cardiovascular, renal, neurológico, metabólico, atividades básicas e intervenções realizadas na assistência ao paciente. É aplicado uma vez ao dia, refletindo as atividades realizadas nas 24 horas anteriores. A pontuação é expressa em porcentagem, variando de 0% a 176,8%, sendo que 100% corresponde à necessidade de um profissional de enfermagem em tempo integral para o paciente^(9,22)^.

A gravidade clínica foi avaliada pelo SOFA, utilizado em UTIs para mensurar o grau de falência orgânica, devendo ser aplicado na admissão e, depois, diariamente^(23,24)^. Avalia seis sistemas: cardiovascular, hepático, respiratório, coagulação, renal e neurológico. Cada um recebe pontuação de 0 a 4, sendo que escores mais altos indicam maior disfunção^(24)^. O SAPS III, composto por 20 parâmetros, é utilizado na admissão em UTI como indicador prognóstico de mortalidade^(25,26)^. Os parâmetros são agrupados em três categorias: condições prévias do paciente, circunstâncias da admissão e disfunção fisiológica^(26)^.

A inclusão dos indicadores de gravidade clínica no instrumento de coleta é justificada por sua relevância para caracterizar a complexidade do cuidado e mensurar demandas assistenciais que influenciam tanto a evolução clínica do paciente quanto a carga de trabalho da equipe de enfermagem. Evidências recentes indicam que escores de gravidade clínica elevados estão associados a uma maior necessidade de intervenções de enfermagem, a uma maior demanda de tempo assistencial e ao aumento do NAS, reforçando a pertinência de integrá-los ao instrumento de coleta e à análise do estudo^(11,12)^. A IRA foi definida conforme o critério creatinina do *Kidney Disease: Improving Global Outcomes* (KDIGO)^(27)^. A gravidade da IRA é estratificada em: KDIGO 1 (risco para lesão renal), quando ocorre aumento da creatinina sérica (Crs) em 0,3mg/dL em 48 horas ou aumento de 1,5 vezes ou 1,9 vezes do valor basal em sete dias; KDIGO 2 (lesão renal), quando o aumento da Crs é 2,0 a 2,9 vezes o valor *baseline;* e KDIGO 3 (falência renal), quando o aumento da Crs é de 3,0 vezes o valor *baseline* ou quando há necessidade de TSR^(27)^. Considerou-se como creatinina basal o menor valor registrado entre sete e 365 dias anteriores à internação na UTI^(3)^.

A doença renal aguda (DRA) foi caracterizada pela persistência da IRA por um período entre sete e 90 dias após o evento desencadeante^(3)^. A recuperação da função renal foi calculada nos pacientes com IRA por meio da razão da Crs no momento da avaliação em relação à Crs basal. Há diferentes níveis de recuperação, como recuperação total da função renal, que ocorre quando a creatinina retorna ao valor da Crs basal^(3)^.

### Tratamento de dados

Na análise estatística, foram calculadas medidas descritivas, tais como média, mediana, desvio padrão, intervalo interquartil, frequência absoluta e porcentagem, para descrição das características das variáveis. O teste qui-quadrado foi utilizado para investigar a associação entre diferentes variáveis categóricas. O teste exato de Fisher foi aplicado quando o tamanho da amostra era pequeno, permitindo avaliar a associação entre duas variáveis categóricas quando as condições de aplicabilidade do teste qui-quadrado não eram atendidas.

Neste estudo, não foi observada normalidade nos dados. Sendo assim, o teste de Wilcoxon-Mann-Whitney foi empregado para comparar as medianas de duas amostras (dependentes ou independentes) em situações em que os dados não atendiam aos pressupostos da distribuição normal e da homogeneidade de variâncias. Na regressão logística, os coeficientes das variáveis independentes foram interpretados em termos de Razão de Chances (*Odds Ratio*), indicando em quanto as chances do evento mudam com um aumento de uma unidade na variável. Para medida da capacidade preditora das variáveis selecionadas do NAS em relação ao tempo de ventilação mecânica, foi confeccionada a curva *Receiver Operator Characteristic* (ROC).

No presente estudo, todas as análises estatísticas foram realizadas utilizando o ambiente de programação R (versão 4.3.2) (*R Core Team,* 2023), e o nível de significância adotado foi de 5%.

### Aspectos éticos

O estudo foi conduzido em conformidade com as diretrizes éticas nacionais e internacionais. Foi aprovado pelo Comitê de Ética em Pesquisa da Universidade de Brasília, Faculdade de Ciências da Saúde, cujo parecer está anexado à presente submissão. O Termo de Consentimento Livre e Esclarecido foi obtido pelos pesquisadores responsáveis junto aos participantes ou quando esses se encontravam impossibilitados de consentir, junto aos seus representantes legais, antes da inclusão no estudo.

## RESULTADOS

Entre os 61 pacientes acompanhados, a média de idade foi de 56 anos, com predominância de indivíduos com mais de 60 anos no grupo com VMP (> 21 dias). A etnia parda foi a mais frequente em todos os grupos, independentemente do tempo de ventilação. Observou-se predominância do sexo masculino na amostra total, especialmente entre os pacientes em VMP. As comorbidades mais prevalentes foram as doenças do sistema circulatório (63%) e endócrinas (52%), com maior frequência no grupo submetido à ventilação mecânica por tempo prolongado.

O uso de DVAs foi significativamente mais frequente no grupo com VMP (p=0,007), com mediana de 17 dias, destacando-se a predominância de drogas vasopressoras (96%) em comparação ao grupo com ventilação inferior a 21 dias (82%). A TSR também foi mais comum entre os pacientes em VMP (70%). O tempo de VMI apresentou diferença estatisticamente significativa, com mediana de 30 dias no grupo com VMP e 13 dias no grupo sem VMP (p<0,001).

A pontuação mediana do SAPS III foi mais elevada no grupo com VMP, com valor de 74. A carga de trabalho da enfermagem, mensurada pelo NAS, apresentou valores elevados em toda a amostra, com mediana de 112% (amplitude interquartil: 106-119), indicando demanda assistencial superior ao tempo integral de um profissional de enfermagem por paciente em 24 horas. Na comparação entre grupos, não foram observadas diferenças estatisticamente significativas entre as pontuações do NAS nos grupos com tempo de ventilação mecânica inferior ou superior a 21 dias. A taxa de óbito intra-hospitalar foi de 41% no grupo em VMP, em comparação a 50% no grupo com ventilação mecânica não prolongada. A IRA grave foi mais frequente no grupo em VMP (78%) em relação ao grupo sem VMP (71%). Tanto a IRA quanto a DRA apresentaram-se mais frequentemente no grupo com VMP (96% e 58%, respectivamente). A recuperação da função renal foi significativamente maior entre os pacientes com ventilação não prolongada (56%) em comparação àqueles com VMP (27%; p=0,034) (Tabela 1).

**Tabela 1:** Caracterização demográfica e clínica de acordo com o tempo (dias) de ventilação mecânica invasiva. Brasília, Distrito Federal, Brasil, 2025

		Ventilação mecânica invasiva		
**Características**	**Total, N = 61**	**≤21, N = 34**	**>21, N = 27**	**Valor de *p***
Idade (anos)				0,079^1^
Média (desvio padrão)	56 (16)	53 (18)	60 (14)	
Mediana (AIQ)	58 [44, 69]	53 [42, 64]	63 [53, 72]	
Sexo masculino, n/N (%)	31/61 (51%)	15/34 (44%)	16/27 (59%)	0,240^2^
Etnia, n/N (%)				0,257^3^
Preta	2/61 (3,3%)	1/34 (2,9%)	1/27 (3,7%)	
Branca	12/61 (20%)	4/34 (12%)	8/27 (30%)	
Indígena	1/61 (1,6%)	1/34 (2,9%)	0/27 (0%)	
Parda	45/61 (74%)	27/34 (79%)	18/27 (67%)	
Asiática	1/61 (1,6%)	1/34 (2,9%)	0/27 (0%)	
Comorbidades				
Cardiopatia, n/N (%)	21/61 (34%)	13/34 (38%)	8/27 (30%)	0,482^2^
Endócrina, n/N (%)	26/61 (43%)	12/34 (35%)	14/27 (52%)	0,194^2^
Circulatória, n/N (%)	31/61 (51%)	14/34 (41%)	17/27 (63%)	0,091^2^
Autoimune, n/N (%)	10/61 (16%)	4/34 (12%)	6/27 (22%)	0,315^3^
Musculoesquelético, n/N (%)	5/61 (8,2%)	3/34 (8,8%)	2/27 (7,4%)	>0,999^3^
Infecções, n/N (%)	10/61 (16%)	3/34 (8,8%)	7/27 (26%)	0,092^3^
Oncohemato, n/N (%)	27/61 (44%)	17/34 (50%)	10/27 (37%)	0,311^2^
Vasopressor, n/N (%)	54/61 (89%)	28/34 (82%)	26/27 (96%)	0,121^3^
Vasodilatador, n/N (%)	4/61 (6,6%)	4/34 (12%)	0/27 (0%)	0,122^3^
Inotrópico, n/N (%)	12/61 (20%)	7/34 (21%)	5/27 (19%)	0,840^2^
Dias totais de DVAs				0,007^1^
Média (desvio padrão)	14 (10)	12 (10)	17 (9)	
Mediana (AIQ)	12 [8, 18]	9 [6, 15]	17 [11, 23]	
SAPS III				0,090^1^
Média (desvio padrão)	70 (16)	67 (18)	74 (11)	
Mediana (AIQ)	73 [59, 80]	68 [52, 77]	74 [65, 82]	
NAS (%)				0,880^1^
Média (desvio padrão)	113 (12)	112 (14)	113 (11)	
Mediana (AIQ)	112 [106, 119]	111,2 [105, 118]	111,8 [107, 119]	
TSR, n/N (%)	38/61 (62%)	19/34 (56%)	19/27 (70%)	0,246^2^
Dias totais de TSR				0,009^1^
Média (desvio padrão)	13 (9)	8 (6)	17 (10)	
Mediana (AIQ)	12 [5, 18]	7 [5, 11]	15 [13, 23]	
Tempo de VMI (dias)				<0,001^1^
Média (desvio padrão)	21 (13)	13 (5)	32 (11)	
Mediana (AIQ)	20 [11, 28]	13 [8, 17]	30 [24, 37]	
Creatinina basal				0,940^1^
Média (desvio padrão)	1,33 (1,23)	1,46 (1,48)	1,15 (0,78)	
Mediana (AIQ)	0,90 [0,60, 1,40]	0,95 [0,60, 1,40]	0,85 [0,70, 1,38]	
Desfecho, n/N (%)				0,179^3^
Alta	1/61 (1,6%)	1/34 (2,9%)	0/27 (0%)	
Entrou em cuidados paliativos	12/61 (20%)	4/34 (12%)	8/27 (30%)	
Óbito	28/61 (46%)	17/34 (50%)	11/27 (41%)	
Transferência para outro hospital	6/61 (9,8%)	2/34 (5,9%)	4/27 (15%)	
Transferência para outro setor	14/61 (23%)	10/34 (29%)	4/27 (15%)	
Óbito intrahospitalar, n/N (%)	28/61 (46%)	17/34 (50%)	11/27 (41%)	0,471^2^
KDIGO, n/N (%)				0,197^3^
0	8/61 (13%)	7/34 (21%)	1/27 (3,7%)	
1	5/61 (8,2%)	2/34 (5,9%)	3/27 (11%)	
2	3/61 (4,9%)	1/34 (2,9%)	2/27 (7,4%)	
3	45/61 (74%)	24/34 (71%)	21/27 (78%)	
IRA, n/N (%)	53/61 (87%)	27/34 (79%)	26/27 (96%)	0,066^3^
DRA, n/N (%)	21/39 (54%)	7/15 (47%)	14/24 (58%)	0,477^2^
Recuperação da função renal, n/N (%)	22/53 (42%)	15/27 (56%)	7/26 (27%)	0,034^2^

Legenda:

1 Teste de soma de postos de Wilcoxon;

2 Teste qui-quadrado de independência;

3 Teste exato de Fisher; AIQ - amplitude interquartil; DVAs - drogas vasoativas; TSR - terapia de substituição renal; VMI - ventilação mecânica invasiva; KDIGO - Kidney Disease: Improving Global Outcomes; IRA - injúria renal aguda; DRA - doença renal aguda; NAS - Nursing Activities Score.

O modo ventilatório assistido-controlado foi o mais utilizado em ambos os grupos. Os parâmetros ventilatórios, assim como os dados laboratoriais da gasometria arterial, não apresentaram diferenças significativas entre os grupos (Tabela 2).

**Tabela 2:** Comparação das variáveis laboratoriais e ventilatórias e o tempo de ventilação mecânica invasiva. Brasília, Distrito Federal, Brasil, 2025

		Ventilação mecânica invasiva		
**Características**	**Total, N = 61**	**≤21, N = 34**	**>21, N = 27**	**Valor de *p***
PEEP média (cm/H2O)				0,217^1^
Média (desvio padrão)	6,81 (1,05)	6,99 (1,20)	6,59 (0,78)	
Mediana (AIQ)	6,62 [6,18, 7,50]	6,72 [6,23, 7,59]	6,53 [6,12, 6,88]	
FIO_2_ média (%)				0,289^1^
Média (desvio padrão)	33 (12)	34 (12)	31 (12)	
Mediana (AIQ)	29 [26, 33]	30 [26, 38]	29 [26, 31]	
Modo ventilatório, n/N (%)				0,283^2^
A/C	53/61 (87%)	28/34 (82%)	25/27 (93%)	
Espontâneo	8/61 (13%)	6/34 (18%)	2/27 (7,4%)	
pH média				0,380^1^
Média (desvio padrão)	7,41 (0,05)	7,40 (0,06)	7,41 (0,04)	
Mediana (AIQ)	7,41 [7,38, 7,43]	7,40 [7,36, 7,43]	7,41 [7,39, 7,43]	
PaO_2_ média (mmHg)				0,149^1^
Média (desvio padrão)	86 (17)	90 (20)	82 (10)	
Mediana (AIQ)	83 [76, 92]	83 [80, 93]	81 [75, 88]	
PaCO_2_ média (mmHg)				0,493^1^
Média (desvio padrão)	37 (8)	37 (7)	38 (8)	
Mediana (AIQ)	36 [32, 40]	36 [32, 40]	36 [33, 40]	
Bicarbonato média (mEq/L)				0,880^1^
Média (desvio padrão)	23,15 (3,82)	23,41 (4,89)	22,82 (1,77)	
Mediana (AIQ)	22,84 [21,55, 24,43]	22,38 [21,12, 25,01]	23,42 [21,92, 24,15]	
Lactato médio (mg/dL)				0,902^1^
Média (desvio padrão)	20 (19)	24 (24)	16 (5)	
Mediana (AIQ)	16 [11, 19]	15 [10, 28]	16 [12, 18]	
Hemoglobina média (g/dL)				0,540^1^
Média (desvio padrão)	8,40 (1,50)	8,45 (1,64)	8,34 (1,34)	
Mediana (AIQ)	8,08 [7,60, 8,86]	8,24 [7,63, 9,13]	8,00 [7,56, 8,60]	

Legenda:

1 Teste de soma de postos de Wilcoxon;

2 Teste exato de Fisher Model; PEEP - pressão positiva no final da expiração; AIQ - amplitude interquartil; FiO_2_ - fração inspirada de oxigênio; SOFA - Sequential Organ Failure Assessment; A/C - assistido-controlado; pH - potencial hidrogeniônico; PaO_2_ - pressão parcial de oxigênio; PaCO_2_ - pressão parcial de gás carbônico.

A análise multivariada indicou que o tempo de uso de DVAs constituiu fator de risco independente para a ocorrência de VMP (p=0,050) (Tabela 3).

**Tabela 3:** Análise multivariada das variáveis clínicas e tempo de ventilação mecânica. Brasília, Distrito Federal, Brasil, 2025

	Univariado		Ajustado		Multivariado	
**Características**	**OR (IC95%)**	**Valor de *p***	**OR (IC95%)**	**Valor de *p***	**OR (IC95%)**	**Valor de *p***
Idade (anos)	1,02 (0,99-1,06)	0,169	1,00 (0,95-1,05)	0,898		
Endócrina	1,64 (0,56-4,88)	0,369	2,18 (0,46-10,6)	0,320		
Circulatória	1,97 (0,68-5,92)	0,217	1,66 (0,37-7,80)	0,509		
Infecções	3,19 (0,78-16,3)	0,123	4,32 (0,71-34,6)	0,131		
Dias totais de DVAs	1,07 (1,01-1,15)	0,050	1,06 (0,99-1,16)	0,124	1,07 (1,01-1,15)	0,050
SAPS III	1,02 (0,98-1,06)	0,285	1,02 (0,96-1,10)	0,451		
SOFA na admissão	1,08 (0,90-1,30)	0,415	0,96 (0,71-1,30)	0,784		
KDIGO	1,30 (0,75-2,45)	0,366	1,20 (0,30-4,51)	0,780		
IRA	4,00 (0,54-81,2)	0,229	2,16 (0,04-194)	0,710		
PaO_2_ média	0,97 (0,92-1,00)	0,134	0,95 (0,88-1,00)	0,055		

*Legenda: OR - Odds Ratio; IC - Intervalo de Confiança; DVAs - drogas vasoativas; SAPS III - Simplified Acute Physiology Score III; SOFA - Sequential Organ Failure Assessment; KDIGO - Kidney Disease: Improving Global Outcomes; IRA - injúria renal aguda; PaO_2_ - pressão parcial de oxigênio.*

Embora os pacientes submetidos à VMP tenham apresentado maior complexidade clínica, caracterizada por maior uso de DVAs, necessidade de TSR e maior frequência de IRA, essas diferenças não se refletiram em aumento da carga de trabalho de enfermagem, conforme demonstrado pela manutenção de pontuações semelhantes de NAS entre os grupos (Figura 2).

**Figura 2:** Comparação dos escores *Simplified Acute Physiology Score*, *Sequential Organ Failure Assessment* e *Nursing Activities Score* com o tempo de ventilação mecânica invasiva em dias. Brasília, Distrito Federal, Brasil, 2025

A gravidade clínica dos pacientes, avaliada pelo SAPS III e pelo SOFA, não apresentou variações significativas em relação ao tempo de VMI. Observou-se que, na admissão, houve elevação do SAPS III e do SOFA no grupo em VMI durante período superior a 21 dias, ainda que, no mesmo período, o NAS não tenha evidenciado diferença entre os grupos. A redução na pontuação do SOFA foi identificada no desfecho, indicando redução da gravidade dos pacientes ao longo do período de ventilação (Figura 2).

Não foi identificada associação significativa entre o tempo de VMI e a carga de trabalho da equipe de enfermagem, mensurada pelo NAS, com *area under the curve* (AUC) de 0,512, indicando que a duração da VMI não influenciou esse desfecho.

## DISCUSSÃO

No presente estudo, não foi observada associação significativa entre o tempo de VMI e a carga de trabalho da equipe de enfermagem em pacientes críticos, mensurada pelo NAS. Embora as medianas do NAS tenham apresentado valores superiores a 100% em ambos os grupos, com VMI inferior a 21 dias e com VMP, indicando elevada demanda assistencial geral, essa diferença não foi estatisticamente significativa. A análise da curva ROC corroborou esse achado, com AUC de 0,512, evidenciando baixo poder discriminatório entre o tempo de ventilação mecânica e a carga de trabalho da equipe de enfermagem. Esses resultados indicam que a duração da VMI, de forma isolada, não foi determinante para o aumento da carga de trabalho mensurada pelo NAS.

Destaca-se que os pacientes submetidos à VMP apresentaram maior complexidade clínica, evidenciada por maior uso de DVAs, maior necessidade de TSR e maior frequência de IRA. No entanto, essa maior gravidade clínica não se refletiu em aumento da carga de trabalho de enfermagem entre os grupos. Esse achado reforça que o NAS mensura o volume e a natureza das intervenções de enfermagem realizadas em um período de 24 horas, não sendo determinado exclusivamente pela gravidade clínica ou pela duração do suporte ventilatório, mas sim pelo conjunto de cuidados efetivamente prestados ao paciente crítico^(28)^.

Evidência indica que uma pontuação de 100 no NAS corresponde à demanda equivalente a um profissional de enfermagem por paciente; entretanto, estudos demonstram que valores superiores a 61 já se associam ao aumento da mortalidade hospitalar^(29)^. No presente estudo, o NAS superior a 100 em ambos os grupos reflete elevada carga assistencial da equipe de enfermagem, independentemente do tempo de ventilação mecânica. Ademais, quase metade dos pacientes evoluiu para óbito intra-hospitalar, evidenciando a gravidade clínica da amostra, corroborada pelos escores prognósticos SAPS III e SOFA, especialmente entre os pacientes em VMP.

A gravidade clínica dos pacientes, expressa pelos escores prognósticos, sugere maior demanda assistencial da enfermagem devido à complexidade das intervenções realizadas na terapia intensiva, como maior necessidade de suporte ventilatório, uso de DVAs, diuréticos e monitorização contínua. Essas intervenções, fundamentais para estabilização clínica, aumentam a complexidade do cuidado e exigem vigilância constante da equipe de enfermagem^(28)^. Contudo, mesmo diante dessas demandas, o NAS permaneceu semelhante entre os grupos, sugerindo que tais cuidados fazem parte da rotina assistencial de pacientes críticos sob ventilação mecânica, independentemente do tempo de permanência nesse suporte^(30,31)^.

Apesar dos avanços nas intervenções terapêuticas, a mortalidade entre pacientes submetidos à VMI permanece elevada, sendo a maior duração do suporte ventilatório associada a piores desfechos clínicos^(29)^. A possibilidade de prever precocemente a duração da VMI pode contribuir para o planejamento de intervenções antecipadas, a otimização da assistência e a redução de custos, além de proporcionar suporte a pacientes e familiares^(32)^. No entanto, os resultados do presente estudo indicam que a carga de trabalho da equipe de enfermagem se mantém elevada mesmo em pacientes com menor tempo de ventilação, reforçando que a sobrecarga assistencial não está restrita à ventilação prolongada.

Estudo de coorte retrospectivo realizado no Uruguai identificou elevada demanda por ventilação mecânica nas UTIs, com VMP em 17% dos pacientes^(33)^. Na presente investigação, 44% dos pacientes em VMI evoluíram para VMP, valor superior descrito na literatura. Esses pacientes, frequentemente classificados como críticos crônicos, apresentam inflamação persistente e maior dependência de terapias de suporte, como TSR e DVAs^(34,35)^. Nesse cenário, o presente estudo confirmou maior necessidade de TSR prolongada no grupo com VMP, refletindo maior gravidade clínica, porém sem associação significativa adicional na carga de trabalho da equipe de enfermagem.

Pacientes mais idosos e com múltiplas comorbidades na admissão apresentam maior predisposição à VMP (por mais de 21 dias)^(34)^. Revisão sistemática conduzida no Paquistão também demonstrou que a presença de multimorbidade está associada ao desenvolvimento de complicações, como IRA, e à evolução para VMP^(36)^. No presente estudo, observou-se elevada frequência de IRA grave entre os pacientes críticos; contudo, a recuperação da função renal foi significativa, especialmente entre aqueles com menor tempo de ventilação, sugerindo manejo clínico adequado.

A VMI é um suporte ventilatório essencial para restauração das trocas gasosas, porém pode induzir ou agravar lesões pulmonares, além de desencadear processos inflamatórios e estresse oxidativo^(37)^. Apesar disso, neste estudo, não foram observadas diferenças significativas nos parâmetros gasométricos entre os grupos ao longo do acompanhamento, indicando estabilidade metabólica semelhante, independentemente do tempo de ventilação. Do ponto de vista da enfermagem, esse achado reforça que a carga de trabalho decorre principalmente das necessidades assistenciais contínuas, como monitoramento, ajuste de parâmetros ventilatórios e cuidados gerais, e não exclusivamente das alterações fisiológicas mensuráveis^(38)^.

Nos cuidados a pacientes em VMI, a enfermagem exerce papel central na implementação de protocolos assistenciais, avaliação contínua do conforto, agitação e níveis de sedação. Essas intervenções exigem observação frequente e tomada de decisão clínica, impactando diretamente a carga de trabalho e contribuindo para elevação do NAS, mesmo em pacientes com menor tempo de ventilação^(39,40)^.

O uso de DVAs foi elevado na amostra estudada, sendo observado em 89% dos pacientes. Estudo realizado na Austrália identificou que 84% dos pacientes críticos em uso de DVAs utilizaram vasopressores^(41)^, achado consoante com o presente estudo. A administração e a titulação dessas drogas demandam monitorização contínua, registros frequentes e avaliações clínicas constantes, o que contribui para o aumento da carga de trabalho da equipe de enfermagem^(42)^.

Diversos medicamentos utilizados na UTI apresentam potencial nefrotóxico, estando associados ao desenvolvimento de IRA^(43)^. No presente estudo, a IRA foi observada em mais da metade dos pacientes, independentemente da duração da ventilação mecânica. A gestão desses pacientes requer vigilância rigorosa, controle hídrico e avaliação laboratorial frequente, atividades que ampliam a demanda de cuidado pela equipe de enfermagem.

Embora evidências sugiram associação entre IRA e maior tempo de ventilação mecânica^(44,45)^, o presente estudo não identificou associação estatisticamente significativa entre essas variáveis, embora tenha sido observada tendência de maior permanência em VMI entre pacientes com IRA. Observou-se, ainda, correlação significativa entre o número total de dias em TSR e o tempo em VMI, refletindo a gravidade clínica e a presença de disfunções multissistêmicas.

A recuperação da função renal foi significativamente maior entre pacientes com menor tempo de ventilação mecânica (p=0,034), achado semelhante ao observado em estudo de coorte histórica, retrospectivo e quantitativo, realizado no Brasil, que associa o desmame precoce da ventilação mecânica à melhora da função renal^(46)^. Esses resultados reforçam a importância do manejo adequado do suporte ventilatório, com potencial associação indireta na carga de trabalho de enfermagem ao longo da internação.

A elevada mortalidade observada no estudo é compatível com a literatura, que associa maior gravidade clínica e disfunção orgânica múltipla a piores desfechos em pacientes submetidos à VMI^(47)^. Os SAPS III e o SOFA se mostraram ferramentas úteis na avaliação prognóstica, com valores mais elevados entre os pacientes em VMP, corroborando achados de outros estudos^(46,47)^.

Os resultados desta pesquisa evidenciam que a carga de trabalho da equipe de enfermagem em pacientes críticos submetidos à ventilação mecânica é elevada independentemente do tempo de ventilação mecânica, reforçando a necessidade de dimensionamento adequado da equipe e planejamento da assistência. A mensuração precisa da carga de trabalho por meio do NAS permite ajustar a relação entre enfermeiros e pacientes às horas efetivas de cuidado, contribuindo para a qualidade da assistência, a segurança dos pacientes e a redução da sobrecarga profissional^(48,49)^.

### Limitações do estudo

As limitações deste estudo incluem o delineamento unicêntrico e o tamanho amostral reduzido, fatores que podem restringir a generalização dos resultados para outros contextos assistenciais. Ademais, embora o NAS contemple as atividades de enfermagem realizadas ao longo de um período de 24 horas, sua aplicação foi realizada uma vez ao dia, o que pode não refletir variações intra-horárias da carga de trabalho. A presença de registros incompletos nos prontuários eletrônicos também pode ter influenciado a precisão da análise do NAS.

### Contribuições para a área da enfermagem, saúde ou políticas públicas

Este estudo contribuiu para ampliação do conhecimento sobre a relação entre a VMI e a carga de trabalho da equipe de enfermagem em UTIs, ao analisar de forma integrada a duração do suporte ventilatório e a mensuração do cuidado por meio do NAS. Ao evidenciar que a carga de trabalho permanece elevada independentemente do tempo de ventilação mecânica, os achados oferecem subsídios para o planejamento assistencial, o dimensionamento adequado da equipe de enfermagem e a qualificação do cuidado a pacientes críticos submetidos à VMP e à ventilação mecânica não prolongada.

## CONCLUSÕES

O tempo de ventilação mecânica não se associou à variação da carga de trabalho da enfermagem, que permaneceu elevada independentemente da duração do suporte ventilatório. A ventilação mecânica esteve relacionada a uma maior gravidade clínica, refletida pela elevada ocorrência de IRA e por escores prognósticos elevados. Esses achados evidenciam que a sobrecarga assistencial da enfermagem em pacientes críticos submetidos à ventilação mecânica decorre principalmente da complexidade clínica e das múltiplas intervenções requeridas, e não exclusivamente do tempo de permanência em ventilação mecânica.

## Data Availability

Os dados de pesquisa disponíveis estão no corpo do artigo.
